# Supplementary material for: Transcriptomic Analysis Insight into the Immune Modulation during the Interaction of Ophiocordyceps sinensis and Hepialus xiaojinensis
Source: Insects. 2022 Dec 5;13(12):1119. doi: 10.3390/insects13121119 (PMC9788539; doi:10.3390/insects13121119)
Supplement: Supplementary file 1 [file insects-13-01119-s001.zip › Table S1.pdf]

Table S1

Sample	Read number	Base number	Mapping ratio	GC content	%＞＝30%
IL1	39,888,179	11,917,515,528	75.31%	46.58%	94.28%
IL2	37,698,632	11,264,582,082	77.99%	49.39%	94.41%
IL3	34,552,138	10,321,724,486	76.04%	45.91%	94.53%
L1	23,618,253	4,770,887,106	82.09%	42.94%	92.04%
L2	15,773,696	3,974,110,122	80.68%	44.36%	94.06%
L3	15,497,932	3,904,705,023	78.38%	44.16%	93.98%

Note: BMK-ID: sample ID used in Biomarker. Read Number: the number of paired-end reads in clean data. Base number: total base number of clean data. The percentage of Mapped Reads in Clean Reads, GC-content: percentage of G,C in clean data. %≥Q30: percentage of bases with Q-score no less than Q30.
