# Supplementary material for: Transcriptomic Analysis Insight into the Immune Modulation during the Interaction of Ophiocordyceps sinensis and Hepialus xiaojinensis
Source: Insects. 2022 Dec 5;13(12):1119. doi: 10.3390/insects13121119 (PMC9788539; doi:10.3390/insects13121119)
Supplement: Supplementary file 1 [file insects-13-01119-s001.zip › Table S11. Primers for qPCR.pdf]

Table S11. A Primers for IL vs. ML

Gene ID	Forward primer	Reverse primer
gene-G6O67_002713	5’-TATTTCCCCGTTGCTGGTCC-3’	5’-AAATTGCCGGCCTTTTCGTC-3’
gene-G6O67_002052	5’-TGTCATATTCGCCTCCGACG-3’	5’-CTACAGCTTCCCCAAGACGG-3’
gene-G6O67_007546	5’-TTGCAAGCAACCAAGTCACG-3’	5’-CGGAACGATTCCGTCTTTGC-3’
gene-G6O67_006216	5’-CGAGGACTATACCACGCGAC-3’	5’-TCCACGGCTAAATCACCGAC-3’
gene-G6O67_003586	5’-CTTGCCAGCTTTGATACCGC-3’	5’-CGTAACGCGAGTCTGGGTAG-3’
gene-G6O67_003659	5’-ATGTTCATGGCCCAGATGCT-3’	5’-TGCTGAAAGCGTCCTCTCAA-3’
gene-G6O67_004613	5’-TCGGGGTGCTACCAAACATC-3’	5’-GCTTCGCCTATCTCGGACTC-3’

Table S11. B Primers for IL vs. L

Gene ID	Forward primer	Reverse primer
BMK_Unigene_081805	5’-CGACAACGGATGGGATGAC-3’	5’-CACCGTAGGCGATCAACTG-3’
BMK_Unigene_047499	5’-AAGGGTTGACAGATGCGGAG-3’	5’-GCCGCTCCGTGAAGATAAG-3’
BMK_Unigene_052152	5’-TGTGGTGGGTCTCAAATGCT-3’	5’-ATGGCTGAGAGGGAAGGTCA-3’
BMK_Unigene_059801	5’-CGCGATTTCTTCACTGCTCG-3’	5’-TGGTAAACGCCTCCTCAACC-3’
BMK_Unigene_036928	5’-TCTCTTCGGTCGCAAAACCG-3’	5’-GGTAGGCAATCAGGTCCTTCC-3’
BMK_Unigene_056259	5’-TATTCTGGAACAACGCGGCT-3’	5’-GAACACACCACTCTCGCTCA-3’
BMK_Unigene_047499	5’-AAGGGTTGACAGATGCGGAG-3’	5’-GCCATATGTGATCCCGCCAT-3’

CK represented the pre-infected larva of *H. xiaojinensis* uninfected. IL represented the one yr post-infected larva of *Hepialus xiaojinensis* by *O. sinensis.* ML represented the mummified larva.
