# Supplementary material for: Transcriptomic Analysis Insight into the Immune Modulation during the Interaction of Ophiocordyceps sinensis and Hepialus xiaojinensis
Source: Insects. 2022 Dec 5;13(12):1119. doi: 10.3390/insects13121119 (PMC9788539; doi:10.3390/insects13121119)
Supplement: Supplementary file 1 [file insects-13-01119-s001.zip › Table S2.pdf]

Table S2

Sample	Read number	Base number	Mapping ratio	GC content	%＞＝30%
IL1	79,776,358	4,168,259	5.22%	46.58%	94.28%
IL2	75,397,264	9,619,535	12.76%	49.39%	94.41%
IL3	69,104,276	3,711,997	5.37%	45.91%	94.53%
ML1	46,803,998	44,014,773	90.04%	60.76%	94.09%
ML2	46,721,190	43,934,540	94.04%	60.47%	93.74%
ML3	44,421,444	41,678,492	93.83%	60.73%	94.37%

Note: Read Number: the number of paired-end reads in clean data. Base number: total base number of clean data. Mapping ratio: The percentage of Mapped Reads in Clean Reads. GC-content: percentage of G,C in clean data. %≥Q30: percentage of bases with Q-score no less than Q30.
